# Health risk assessment of volatile organic compounds exposure near Daegu dyeing industrial complex in South Korea

**DOI:** 10.1186/s12889-018-5454-1

**Published:** 2018-04-20

**Authors:** Jianfei Shuai, Sunshin Kim, Hyeonsu Ryu, Jinhyeon Park, Chae Kwan Lee, Geun-Bae Kim, Venecio U. Ultra, Wonho Yang

**Affiliations:** 1 Department of Occupational Health, College of Public Health, Daegu Catholic University, Hayang-eup, Gyeongsan-si, Gyeongbuk 712-702 South Korea; 2 College of Public Health, Xinxiang Medical University, Xinxiang, China; 3 Environmental Health Center for Hazardous Gas Exposure, Gumi Hospital, Soonchunhyang University College of Medicine, Gumi, Korea; 4 Department of Occupational and Environmental Medicine, Inje University, Gimhae, South Korea; 5 Environmental Health Research Division, National Institute of Environmental Research, Seo-gu, South Korea; 6 Department of Earth and Environmental Sciences, Botswana International University of Science and Technology, Palapye, Botswana

**Keywords:** Air pollutants, Dyeing industry, Exposure, Health risk

## Abstract

**Background:**

Studying human health in areas with industrial contamination is a serious and complex issue. In recent years, attention has increasingly focused on the health implications of large industrial complexes. A variety of potential toxic chemicals have been produced during manufacturing processes and activities in industrial complexes in South Korea. A large number of dyeing industries gathered together in Daegu dyeing industrial complex. The residents near the industrial complex could be often exposed to volatile organic compounds. This study aimed to evaluate VOCs levels in the ambient air of DDIC, to assess the impact on human health risks, and to find more convincing evidences to prove these VOCs emitted from DDIC.

**Methods:**

According to deterministic risk assessment, inhalation was the most important route. Residential indoor, outdoor and personal exposure air VOCs were measured by passive samplers in exposed area and controlled area in different seasons. Satisfaction with ambient environments and self-reported diseases were also obtained by questionnaire survey. The VOCs concentrations in exposed area and controlled area was compared by t-test. The relationships among every VOC were tested by correlation. The values of hazard quotient (HQ) and life cancer risk were estimated.

**Results:**

The concentrations of measured VOCs were presented, moreover, the variety of concentrations according the distances from the residential settings to the industrial complex site in exposed area. The residential indoor, outdoor, and personal exposure concentrations of toluene, DMF and chloroform in exposed area were significantly higher than the corresponding concentrations in controlled area both in summer and autumn. Toluene, DMF, chloroform and MEK had significantly positive correlations with each other in indoor and outdoor, and even in personal exposure. The HQ for DMF exceeded 1, and the life cancer risk of chloroform was greater than 10^− 4^ in exposed area. The prevalence of respiratory diseases, anaphylactic diseases and cardiovascular diseases in exposed area were significantly higher than in controlled area.

**Conclusions:**

This study showed that adverse cancer and non-cancer health effects may occur by VOCs emitted from DDIC, and some risk managements are needed. Moreover, this study provides a convenient preliminarily method for pollutants source characteristics.

## Background

Air pollution continues to receive a great deal of interest worldwide due to its negative impacts on economic losses and human health. Industrial complexes may be important anthropogenic sources of air pollution. Studies show links between living near the industrial complexes and occurrences of adverse health outcomes [1, 2]. How living near industrial complexes contributes to poor air quality and adverse health outcomes is an ongoing concern [3].

Daegu Dyeing Industrial Complex (DDIC) was established in Daegu city in 1980. Daegu is the 3rd largest city with about 3.5 million people in South Korea. The area of DDIC is 859,929 square meters, and it includes about 117 factories and 8414 employees in total. Factories in DDIC specialize in textile dyeing and printing. The dyeing process is characterized by the high consumption of water, fuel and a variety of chemicals in a long process that generates an immense amount of waste. According to Pollutant Release and Transfer Registers of the Ministry of Environment (PRTR), a institution to collect and disseminate information on environmental releases and transfers of chemical substances from industries and other facilities found out that an average of 56.93 tons of pollutants was emitted into the ambient air per year from DDIC during the years 2005 ~ 2011. One of the main environmental problems is air pollutants emissions such as volatile organic compounds (VOCs).

Volatile organic compounds are an important group of air pollutants, which are often referred to as toxic or hazardous air pollutants (HAPs) [4]. They play an important role in the formation of ozone and fine particulate matter by photochemical smog [5–7], and also contribute to most serious health-related impacts. They also cause acute symptoms such as irritations of the nose, throat, and eyes, cause headaches, nausea, dizziness, allergic skin reactions, and can also damage the internal organs such as the liver and kidneys. Moreover, some compounds of VOCs may not be immediate hazards but can lead to chronic health risks. Toluene and xylene could result in serious neurosis [8–11]. Chronic toluene exposure leads to devastating neurological disorders, of which dementia is the most serious [12, 13]. Long-term exposure to xylene may cause headaches, extreme tiredness, tremors, impaired concentration and short-term memory [14]. Chronic exposure to chloroform by inhalation in human is associated with effects on the liver and central nervous system including hepatitis, jaundice, depression, and irritability [15]. Based on above, the hypotheses of this study were established as residents near DDIC were exposed to high levels of VOCs, moreover, health would be affected. Therefore, this study aimed to evaluate the levels of VOCs in the ambient air near DDIC and to assess the exposure levels and possible risks for the residents near DDIC. Moreover, this study also tried to find more convincing pieces of evidence to prove that ambient VOCs were emitted from DDIC.

## Methods

### Study sites

**Fig. 1:**
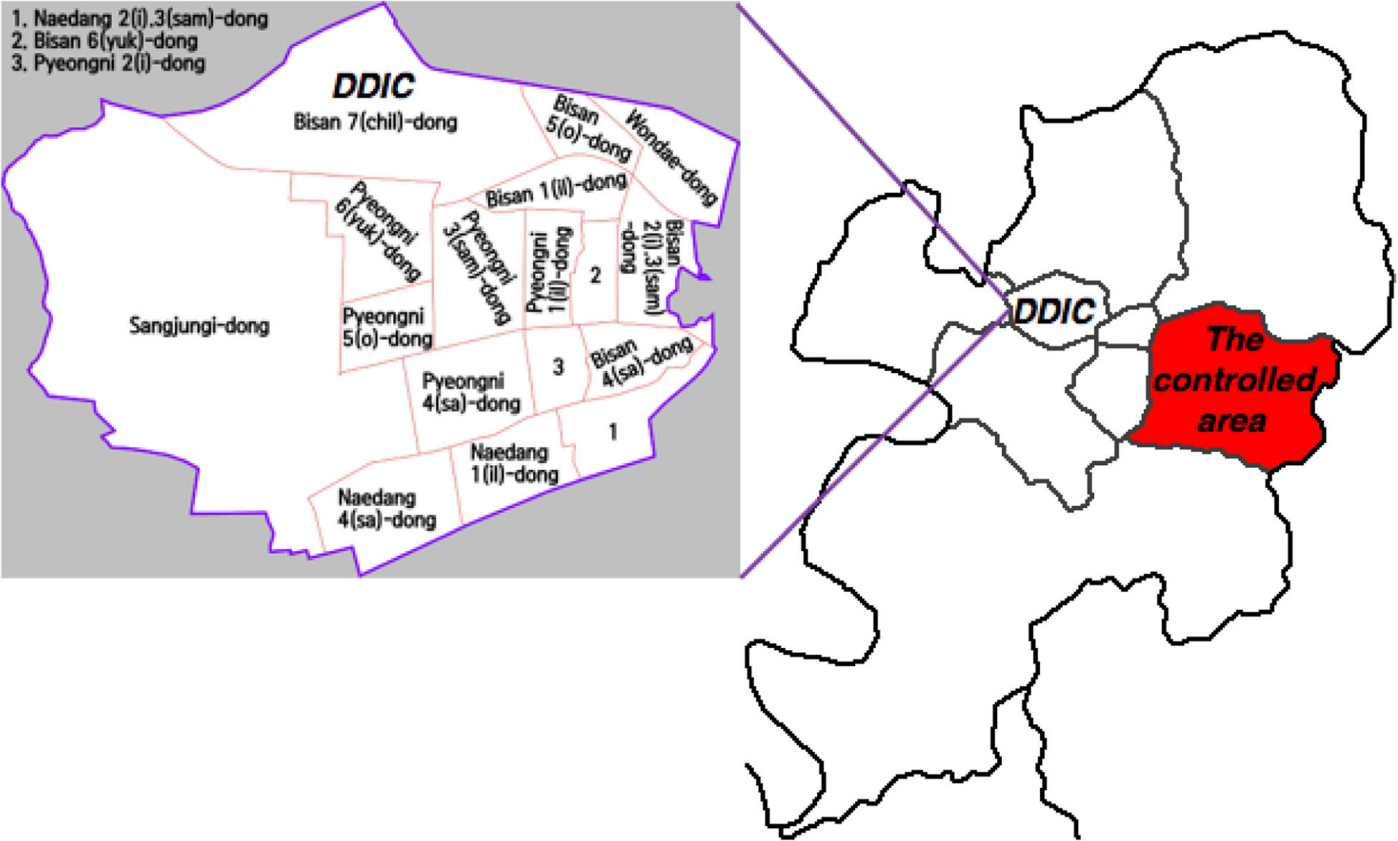
Locations of the exposed area and controlled area. Description: Locations of the exposed area and controlled area were shown. And the administrative unit in exposed area was also shown

Daegu Dyeing Industrial Complex located in the Seogu district of Daegu was the exposed area. It is located in western Daegu with an area of 17.48 km^2^ (Fig. 1). Its population was 215,399 in 2013, and among them, 50.70% were males and 49.35% were females. The aged population, those more than 65 years old accounted for 12.90% of the total population. There were 369 pollutant emission sources in the exposed area, however, DDIC was the main area source. Suseonggu district was selected as the controlled area for contrast, which was 10 km away from the exposed area. It had a population of 460,714 in 2013. The proportion of male and female in the controlled area and exposed area was similar. The aged population in the controlled area (10.80%) was less than that of the exposed area. The controlled area was the political and cultural center of Daegu, with less factories than those in the exposed area. There were 14 pollutant emission sources in the controlled area.

Daegu has a continental climate with dry winters and hot summers. The area receives little precipitation except during the rainy season of summer. According to the Korea Meteorological Administration, the average precipitation varies from 224.0 mm to 235.9 mm between July and August (summer). On the contrary, the average precipitation varies from 33.8 mm to 30.5 mm between October and November (autumn). Over a year, the temperature of Daegu typically varies from − 5 °C to 32 °C with 9.0 °C of daily mean temperature. Southeast wind prevails in July and August, while it is the northwest in October and November.

### Study design and participants

This is a cross-sectional study of ambient airborne chemicals originating from dyeing industry complex and their adverse health effects to populations nearby. The study focused primarily on eight principal air pollutants: toluene, dimethylformamide (DMF), chloroform, methylethylketone (MEK), benzene, ethylbenzene, *m,p*-xylene and *o*-xylene. Toluene, DMF, chloroform, and MEK were the main air pollutants emitted by DDIC according to pollutants emissions data from PRTR. However, these compounds were also known as markers of vehicle emissions [16, 17]. While benzene was not utilized in dyeing and finishing processes, it was also selected as a marker of vehicle emissions for evaluating the potential influence on gasoline vehicle emissions [18]. Benzene content was 1% ∼ 3% in unit volume of gasoline in South Korea, according to the South Korean Petroleum Corporation.

A total of 1261 volunteers from 822 families in the exposed area and 449 volunteers from 212 families in the controlled area participated in this study. Volunteers were selected by stratified random sampling method according every 500 m from the residential settings to DDIC. Smokers were excluded considering smoking as a confounding factor. One male and one female were both chosen from each household where it was possible. The ratio of volunteers in the exposed area and the controlled area was about 3:1. A questionnaire administered by a trainer interviewer was filled out by each participant. Questions include information related to demographic characteristics, residential environments, personal habits such as satisfaction with ambient environments and histories of diseases. Other information regarding the influencing factors was also surveyed, such as the use of air fresheners, recent painting, and proximity to busy roads. The respondents’ satisfaction with ambient environments was determined by choosing one from the following descriptions of their feelings: very satisfied, satisfied, ordinary, dissatisfied and extremely dissatisfied. Their health status was included by finding out if medical diagnoses were made about respiratory, anaphylactic and cardiovascular diseases in the last 6 months. The questionnaire survey was conducted simultaneously in the exposed and controlled area in July 2013. The volunteers were blinded to the purpose of this study. This study was approved by the Medical Center Institutional Review Board in Daegu Catholic University (CR-13-053-RES-001-R) on June 10th, 2013.

Measurement of VOCs immediately followed the questionnaire survey, where 310 volunteers from 127 families in the exposed area, and 150 volunteers from 59 families in the controlled area participated. These volunteers were also enrolled from the volunteers participated in questionnaire survey according to the same methods. Residential indoor, outdoor and personal VOCs concentrations were simultaneously measured from Monday to Friday by passive samplers (3 M, USA, OVM #3500). The measurements were conducted two times: summer (July–August) and autumn (October–November) in 2013. Residential indoor and outdoor passive samplers were put at the heights of human respiration zone in indoor and outdoor microenvironments. Passive samplers were placed to avoid possible VOCs sources or ventilation vents such as doors and windows. Outdoor passive samplers were protected from rain and direct sunlight. All volunteers were asked to carry passive samplers at their breathing zones while awake and to place samplers near breathing zones while asleep. The measurements of VOCs concentrations were also conducted simultaneously in the exposed and controlled area. Field blanks and 15% duplicate measurements were conducted for quality control. The mean relative standard deviations for the measured compounds were all less than 15%.

After taking the measurements, the passive samplers were carefully packaged for airproofing. Then, the samplers were sent to the laboratory immediately and were stored in the refrigerator at 4 °C. After all the samples were collected, they were analyzed. Sorbent in the sampler was transferred to a vial, and 1.5 mL carbon disulphide (CS_2_) was added to the vial subsequently. The vial was placed to stand for 45–60 min with occasional stirring for desorption. The extract was then sealed in auto-sampler vials and analyzed by Gas Chromatograph-Mass Selective Detector (GC-MS, Clarus SQ 8 T, Perkin Elmer). The limit of detection (LOD) was calculated as three times the standard deviation of the seven field blanks. Half the LOD for each compound was used in the analyses for samples in which a certain compound was not detected.

### Human risk estimation

Long-term health effects of exposure to target VOCs were estimated. Based on the Guidance on Risk Assessment for Air Contaminant Emissions [19], hazard quotient (HQ) was used to characterize health risk for non-carcinogens. Inhalation health hazard quotient was defined by following the equation for long-term exposure:

$$ \mathrm{HQ}=\frac{C_{air}}{RfC} $$

Where, C_air_: Concentration of pollutant in air, μg/m^3^, RfC: pollutant-specific reference concentration, μg/m^3^

Human health risk estimates for inhalation of carcinogens are based on the following calculation:

$$ \mathrm{Cancer}\ \mathrm{risk}=\mathrm{C}\times \mathrm{URF} $$

Where: C = ambient air concentration of a pollutant, μg/m^3^, URF = pollutant-specific inhalation risk factor, (μg/m^3^)^− 1^

The values of URF and RfC were obtained from the Integrated Risk Information System (IRIS) and the California Office of Environmental Health Hazard Assessment (OEHHA). Risk assessments were carried out for personal exposure concentrations using 50% and 95% percentiles in both the exposed area and the controlled area. The 50% percentile concentrations were used to represent the main group of individuals and the 95% percentile concentrations represented the highest exposure group in the volunteers. For non-carcinogens, a reference value of less than or equal to unity (HQ ≤ 1) was taken as the acceptable risk. For HQ values greater than unity (HQ > 1), the higher the value, the greater is the likelihood of adverse non-carcinogenic health impacts. For carcinogens, incremental risks between 10^− 4^ and 10^− 7^ are generally perceived as being reasonable and adequate for the protection of human health. In reality, however, populations may be exposed to the same constituents from other sources which are unknown or unrelated to a specific study. Therefore, it is preferable that the estimated carcinogenic risk is well below the 10^− 6^ benchmark level to allow for a reasonable margin of protectiveness for populations at potential risk. Indeed, if a calculated cancer risk exceeds the 10^− 6^ benchmark, the need for corrective measures and risk management actions require serious consideration.

### Statistical methods

The statistical analyses were performed using the SPSS for Windows Versions 22 (SPSS, Chicago, IL). The Kolmogorov-Smirnov test was used to evaluate the normality of the data. The satisfaction degrees with ambient environments and prevalence of diseases obtained through the questionnaires were tested using chi-square tests. The comparisons were tested using t-tests between the VOCs concentrations in the exposed area and the controlled area. The relationships of indoor, outdoor and personal VOCs were tested by Pearson correlation. The significance level was set to *p* < 0.05 in all analyses.

## Results

The compositions of respondents for the questionnaire survey were similar for both the exposed and the controlled areas. Female respondents were dominant both in the exposed (61.10%) and the controlled (54.60%) areas. Respondents aged 50 years to 59 years were the most numbered both in the exposed area (31.50%) and in the controlled area (38.10%), followed by the respondents aged 40 years to 49 years. Respondents aged 40 years to 49 years were about 19.70% in the exposed area and 17.40% in the controlled area, respectively. Respondents aged 60 years to 69 years in the exposed area (15.00%) were more than those in the controlled area (9.80%). On the contrary, the young respondents (aged from 20 years to 29 years) in the exposed area (8.40%) were less than those in the controlled area (15.80%).

The questionnaire survey reported extremely severe or severe foul odor by 53.37% of the respondents in the exposed area as shown in Table 1. Thought perception about ambient air pollution was extremely severe or severe and was held by 57.96% of the respondents in the exposed area. On the contrary, serious or extremely serious odor was reported by only 4.64% of those in the controlled area. There were 51.76% of the respondents in the exposed area who did not satisfy their ambient environments. However, only 4.99% of the respondents were not satisfied with their ambient environments in the controlled area, while 53.32% of the respondents in the exposed area regarded that living at DDIC would affect their health. The subjects who lived in the exposed area reported environmental dissatisfaction more frequently than the subjects who lived in the controlled area.

**Table 1:** Perception level about ambient environments and prevalence of diseases diagnosed by the questionnaire survey

	Degree	Exposed group		Controlled group		Trend
		*N*	Percent	*N*	Percent	*p*
*Satisfaction with ambient environment*						
Foul odor	Extremely serious	347	28.23%	1	0.23%	< 0.01
	Serious	309	25.14%	19	4.41%	
	Normal	474	38.57%	223	51.74%	
	Not serious	84	6.83%	151	35.03%	
	No problem	15	1.22%	37	8.58%	
Ambient air Pollution	Extremely serious	356	29.06%	7	1.59%	< 0.01
	Serious	354	28.90%	40	9.11%	
	Normal	457	37.31%	244	55.58%	
	Not serious	52	4.24%	122	27.79%	
	No problem	6	0.49%	26	5.92%	
Satisfaction with ambient environment	Extremely dissatisfied	300	24.61%	3	0.68%	< 0.01
	Dissatisfied	331	27.15%	19	4.31%	
	Normal	481	39.46%	196	44.44%	
	Satisfied	105	8.61%	192	43.54%	
	Very satisfied	2	0.16%	31	7.03%	
Health impacts of DDIC	No	200	16.21%	202	45.70%	< 0.01
	Normal	376	30.47%	139	31.45%	
	Slight	289	23.42%	66	14.93%	
	Extremely serious	369	29.90%	35	7.92%	
*History of diseases*						
Asthma		46	3.65%	7	1.56%	0.03
Anaphylactic rhinitis		141	11.18%	35	7.80%	0.04
Allergic conjunctivitis		62	4.92%	12	2.67%	0.05
Cardiovascular diseases		44	3.49%	5	1.11%	0.01
Alveobronchiolitis		56	4.44%	1	0.22%	< 0.01
Allergic dermatitis		75	5.95%	11	2.45%	< 0.01
Thyroid diseases		61	4.84%	10	2.23%	0.02
Cancers		30	2.40%	4	0.90%	0.05

The distribution of self-reported diseases among those in the exposed area and the controlled area was also shown in Table 1. Anaphylactic rhinitis was significantly more prevalent in the exposed area compared to the controlled area (*p* < 0.05). There were 5.95% of the respondents in the exposed area who reported allergic dermatitis, and 2.45% in the controlled area (*p* < 0.01). The prevalence of alveobronchiolitis was 4.44% in the exposed area and was 0.22% in the controlled area (*p* < 0.01). Diseases like asthma, cardiovascular diseases and thyroid diseases were more prevalent in the exposed area than in the controlled area (*p* < 0.05).

The main air pollutants emitted from DDIC and their emissions data during the years 2005~ 2011 were shown in Table 2. The collective amounts of toluene, DMF, chloroform, and MEK accounted for 92.24% of the total air emissions in the seven-year period. Especially, the mean amount of toluene was 344,552 kg. The second major air pollutant was DMF, which was 81,441 kg. The average quality of chloroform and MEK emitted into the air was 65,241 kg and 33,913 kg, respectively. The other air pollution emissions were relatively small.

**Table 2:** The main atmospheric emissions inventory of DDIC from 2005 ~ 2011 (Unit: kg)

Pollutant	CAS No.	2005	2006	2007	2008	2009	2010	2011	Total
Toluene	000108–88-3	370,079	364,082	360,505	350,988	245,290	408,481	312,438	2411,863
DMF	000068–12-2	68,419	85,167	81,191	95,090	88,684	80,502	71,033	570,087
Chloroform	000067–66-3	61,559	68,741	70,854	67,929	80,157	74,593	32,851	456,684
MEK	000078–93-3	22,180	31,619	30,825	32,374	36,362	37,993	46,041	237,394
Carbon tetrachloride	000056–23-5	13,994	49,016	49,579	13,857	11,494	10,379	7622	155,941
Hydrogen peroxide	007722–84-1	5636	4363	17,433	23,359	16,403	23,998	2511	93,703
Acetic acid	000064–19-7	7083	7252	5800	5013	7789	9076	6924	48,937
Sodium hydroxide	001310–73-2	1582	2323	1118	1095	576	1252	0	7947
Hydrogen chloride	007647–01-0	188	254	231	216	245	227	228	1589
Ammonia	007664–41-7	82	113	155	102	125	129	0	706
Hydrazine hydrate	007803–57-8	0	0	0	0	0	246	236	482
Nickel and compounds	007440–02-0	0	0	0	10	5	0	0	15
Chrome and compounds	007440–47-3	0	0	0	3	10	0	0	13
Sulfuric acid	007664–93-9	0	8	0	0	0	0	0	8
Total		550,802	612,938	617,691	590,036	487,140	646,876	479,884	3,985,369

The measured indoor and outdoor concentrations of selected VOCs were shown in Table 3. Residential outdoor and indoor average concentrations of toluene, DMF, chloroform, benzene, ethylbenzene and *m,p*-xylene in the exposed area were significantly higher than in the controlled area in summer. The outdoor and indoor average concentrations of most selected VOCs in the exposed area were also significantly higher than in the controlled area in autumn, except for MEK and *m, p*-xylene. Specifically, the average outdoor concentration of toluene in the exposed area was 73.62 μg/m^3^ in summer and 161.37 μg/m^3^ in autumn, respectively.

**Table 3:** Indoor and outdoor VOCs concentrations in exposed and controlled area (Unit: μg/m^3^)

		Summer				Autumn			
		Exposed area (*N* = 310)	Controlled area (*N* = 150)	Exposed area/Controlled area	*p* value	Exposed area (*N* = 310)	Controlled area (*N* = 150)	Exposed area/Controlled area	*p* value
		Mean ± SD	Mean ± SD			Mean ± SD	Mean ± SD		
Toluene	Out^a^	73.62 ± 62.07	11.38 ± 13.67	6.47	< 0.01	161.37 ± 211.73	74.67 ± 51.81	2.16	< 0.01
	In^b^	125.22 ± 396.86	16.81 ± 26.75	7.45	0.03	167.04 ± 221.10	72.51 ± 56.62	2.30	< 0.01
DMF	Out	23.17 ± 28.60	4.79 ± 3.05	4.84	< 0.01	21.26 ± 20.30	3.40 ± 1.20	6.25	< 0.01
	In	21.30 ± 33.12	4.90 ± 3.12	4.35	< 0.01	17.62 ± 15.41	3.59 ± 1.03	4.91	< 0.01
Chloroform	Out	10.75 ± 4.13	5.77 ± 4.63	1.86	< 0.01	11.72 ± 5.75	6.71 ± 1.66	1.75	< 0.01
	In	10.38 ± 3.44	6.47 ± 4.85	1.60	< 0.01	11.92 ± 5.78	6.70 ± 2.21	1.78	< 0.01
MEK	Out	2.67 ± 6.67	N.D.^c^	N.D.	N.D.	13.84 ± 10.54	15.42 ± 56.46	0.90	0.33
	In	3.03 ± 7.15	N.D.	N.D.	N.D.	15.15 ± 13.89	30.65 ± 138.31	0.49	0.33
Benzene	Out	8.84 ± 2.15	5.47 ± 3.45	1.62	< 0.01	3.54 ± 2.54	1.85 ± 0.41	1.91	< 0.01
	In	9.00 ± 2.02	5.51 ± 3.43	1.63	< 0.01	3.71 ± 2.93	1.94 ± 0.52	1.91	< 0.01
Ethylbenzene	Out	20.38 ± 22.66	5.89 ± 5.81	3.46	< 0.01	2.34 ± 1.42	1.27 ± 0.78	1.84	< 0.01
	In	20.23 ± 16.63	6.22 ± 6.78	3.25	< 0.01	2.62 ± 14.79	1.57 ± 2.31	1.67	0.02
*m,p*-Xylene	Out	10.70 ± 10.10	3.51 ± 2.57	3.05	< 0.01	10.48 ± 3.55	15.37 ± 7.95	0.68	< 0.01
	In	12.11 ± 10.94	3.81 ± 2.61	3.18	< 0.01	11.13 ± 4.14	18.69 ± 17.25	0.60	0.16
*o*-Xylene	Out	17.42 ± 7.92	9.23 ± 5.87	1.89	0.16	1.73 ± 0.48	N.D.	N.D	N.D
	In	18.09 ± 8.03	9.94 ± 7.16	1.82	0.01	1.82 ± 0.51	N.D.	N.D	N.D

Residential outdoor and indoor average concentrations of toluene, DMF, chloroform, benzene, ethylbenzene and *m,p*-xylene in exposed area were significantly higher than in the controlled area in summer. The outdoor and indoor average concentrations of most selected VOCs in exposed area were also significantly higher than in the controlled area in autumn, only except for MEK and *m, p*-xylene

^a^Outdoor; ^b^Indoor; ^c^No detection

The average outdoor concentration of toluene in the exposed area was 6.47 times higher than in the controlled area. Moreover, the indoor concentration of toluene in the exposed area was 7.45 times higher than in the controlled area. Both outdoor and indoor concentrations of DMF and ethylbenzene in the exposed area were more than 3 times higher than in the controlled area. The personal concentrations of VOCs were shown in Table 4. The personal concentrations of toluene, DMF, chloroform, benzene and *m,p*-xylene in the exposed area were significantly higher than the corresponding personal concentrations in the controlled area in summer. The personal concentrations of toluene, DMF, chloroform, and benzene in the exposed area were also significantly higher than that in the controlled area in autumn. The relationships of personal concentrations in the exposed and the controlled area were in accordance with the concentrations of indoor and outdoor in the two areas.

**Table 4:** Personal VOCs concentrations in the exposed and the controlled areas (Unit: μg/m^3^)

	Exposed area (*N* = 310, Mean ± SD)	Controlled area (*N* = 150, Mean ± SD)	Exposed area / Controlled area	*p*-value
*Summer*				
Toluene	125.59 ± 230.50	27.47 ± 53.83	4.57	< 0.01
DMF	24.14 ± 41.54	5.63 ± 4.57	4.29	0.01
Chloroform	9.03 ± 4.61	6.05 ± 4.36	1.49	< 0.01
MEK	4.23 ± 12.34	N.D.^a^	N.D.	N.D.
Benzene	10.22 ± 6.76	6.06 ± 4.25	1.69	< 0.01
Ethylbenzene	17.95 ± 27.08	10.46 ± 22.17	1.72	0.11
*m,p*-Xylene	13.38 ± 14.83	6.55 ± 9.50	2.04	< 0.01
*o*-Xylene	19.12 ± 15.12	11.03 ± 7.73	1.73	0.73
*Autumn*				
Toluene	171.59 ± 232.57	89.21 ± 77.02	1.92	< 0.01
DMF	21.11 ± 21.26	3.62 ± 1.06	5.83	< 0.01
Chloroform	11.18 ± 5.63	7.31 ± 2.17	1.53	< 0.01
MEK	13.73 ± 12.10	31.74 ± 98.05	0.43	0.20
Benzene	3.58 ± 2.98	2.04 ± 0.64	1.75	< 0.01
Ethylbenzene	2.42 ± 1.59	1.98 ± 2.44	1.22	0.09
*m,p*-Xylene	10.44 ± 3.65	22.59 ± 17.72	0.46	< 0.01
*o*-Xylene	1.74 ± 0.41	6.80 ± 5.69	0.26	0.01

^a^Not Detected

The relationships among indoor and outdoor concentrations of the eight selected VOCs in the exposed area were also analyzed in Table 5. The indoor concentrations of all measured VOCs were positively correlated with outdoor concentrations in the exposed area (*p* < 0.01). Mutual positive correlations were significant among toluene, DMF, chloroform, and MEK both in the residential indoor and outdoor. Benzene, ethylbenzene, *m,p*-xylene, and *o*-xylene also showed mutual positive correlation both in the residential outdoor and indoor. However, correlations were not significant between the two groups of VOCs.

**Table 5:** Spearman correlation between each indoor and outdoor concentration of target VOCs in exposed area (*N* = 310)

	Chloroform^b^	Chloroform^a^	MEK^b^	MEK^a^	Benzene^b^	Benzene^a^	Toluene^b^	Toluene^a^	Ethylbenzene^b^	Ethylbenzene^a^	*m,p*-Xylene^b^	*m,p*-Xylene^a^	*o*-Xylene^b^	*o*-Xylene^a^	DMF^b^
Chloroform^a^	0.42**	1													
MEK^b^	0.26*	0.27*	1												
MEK^a^	0.24*	0.34**	0.60**	1											
Benzene^b^	0.45**	0.13	−0.14	−0.14	1										
Benzene^a^	0.26**	0.27**	−0.17	0.03	0.78**	1									
Toluene^b^	0.42**	0.24**	0.56**	0.43**	0.14	0.08	1								
Toluene^a^	0.15	0.32**	0.41**	0.66**	−0.05	0.19*	0.67**	1							
Ethylbenzene^b^	0.45**	0.10	−0.01	−0.02	0.39**	0.26**	0.36**	0.12	1						
Ethylbenzene^a^	0.13	0.33**	0.15	0.21	0.08	0.29**	0.26**	0.46**	0.52**	1					
*m,p*-Xylene^b^	0.29**	0.14	0.08	−0.12	0.49**	0.43**	0.22**	0.01	0.58**	0.31**	1				
*m,p*-Xylene^a^	0.08	0.16	0.21	0.22*	0.14	0.36**	0.18	0.44**	0.16	0.56**	0.39**	1			
*o*-Xylene^b^	0.46**	0.18	−0.08	−0.05	0.76**	0.65**	0.15	0.02	0.66**	0.34**	0.71**	0.34**	1		
*o*-Xylene^a^	0.28**	0.38**	0.06	0.10	0.52**	0.64**	0.16	0.30**	0.36**	0.72**	0.48**	0.63**	0.67**	1	
DMF^b^	0.39**	0.31**	0.55**	0.40**	0.05	0.05	0.73**	0.48**	0.06	0.02	0.03	0.07	−0.03	0.04	1
DMF^a^	0.10	0.32**	0.41**	0.36**	0.20*	−0.01	0.44**	0.56**	−0.25**	−0.02	− 0.20*	0.10	− 0.24**	−0.09	0.66**

The indoor concentrations of all measured VOCs were positively correlated with outdoor concentrations in the exposed area (*p* < 0.01). Positive mutual correlations were significant among toluene, DMF, chloroform, and MEK both in residential indoor and outdoor

^a^Indoor; ^b^Outdoor; *p < 0.05; **p < 0.01

The relationships of the personal VOCs concentrations in the exposed area were shown in Table 6. Positive mutual correlations were also significant among personal exposure concentrations of toluene, DMF, chloroform, and MEK. Personal exposure concentrations of benzene, ethylbenzene, *m,p*-xylene, and *o*-xylene also showed mutual positive correlation Correlations were not significant between the two groups. Significant positive correlations in the group of toluene, DMF, chloroform and MEK and the group of benzene, ethylbenzene, *m,p*-xylene, and *o*-xylene were in accordance with outdoor and indoor concentrations.

**Table 6:** Spearman correlations between concentrations of personal VOCs exposure in exposed area (*N* = 310)

	Chloroform	MEK	Benzene	Toluene	Ethylbenzene	*m,p*-Xylene	*o*-Xylene
MEK	0.27**	1					
Benzene	0.60**	0.17	1				
Toluene	0.33**	0.75**	0.12	1			
Ethylbenzene	0.39**	0.05	0.19*	0.17*	1		
*m,p*-Xylene	0.44**	0.06	0.31**	0.18*	0.80**	1	
*o*-Xylene	0.46**	0.03	0.25**	0.06	0.80**	0.89**	1
DMF	0.27**	0.27**	0.09	0.62**	0.14	0.22**	0.07

*: *p* < 0.05; **: *p* < 0.01

**Fig. 2:**
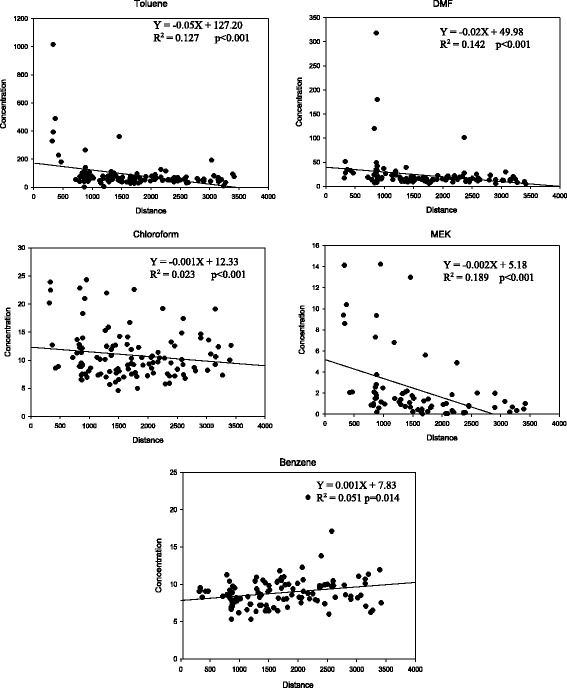
Relationships between outdoor VOCs concentrations and distances from DDIC in exposed area (Concentration: μg/m^3^, Distance: metre). Description: The formula referred to the variation of outdoor VOC concentration with the distance from DDIC increased. Figure. 1 showed the outdoor concentrations of toluene, DMF, chloroform, and MEK in the exposed area significantly decreased with the distances increased from measuring sites to the site of DDIC. Conversely, the concentration of outdoor benzene in the exposed area increased with the distances increased from measuring sites to the site of DDIC

Relationships between outdoor VOCs concentrations in exposed area and distances from measuring sites to DDIC were shown in Fig. 2. The outdoor concentrations of toluene, DMF, chloroform and MEK significantly decreased with the distances increased from DDIC. Conversely, the concentration of outdoor benzene was found to increase with distances increased from DDIC. The decreasing trends of outdoor concentrations of ethylbenzene, *m,p*-xylene, and *o*-xylene were also shown; however, it was not significant (*p* > 0.10).

The hazard quotients (HQ) of toluene, MEK, *m,p*-xylene and *o*-xylene did not exceed 1 both in the exposed area and the controlled area as shown in Table 7. The hazard quotient of DMF exceeded 1 in the exposed area using concentration of 95% percentile. Concerning carcinogens, both cancer risks of chloroform and ethylbenzene were all greater than one in million (10^− 6^). Although cancer risks of chloroform were greater than one in ten thousand (10^− 4^) both in the exposed area and controlled area, cancer risk in the exposed area was much greater than in the controlled area.

**Table 7:** Health risk estimates for inhalation of VOCs in the exposed and the controlled areas (*N* = 310, Unit: μg/m^3^)

	Concentration (50% percentiles)	HQ or Cancer risk	Concentration (95% percentiles)	HQ or Cancer risk
*Non-carcinogens*				
Toluene^a^	94.48	0.02	721.49	0.14
Toluene^b^	48.05	0.01	207.20	0.04
MEK^a^	9.45	0.18E-2	34.81	0.01
MEK^b^	9.63	0.19E-2	20.72	0.40E-2
*m,p*-Xylene^a^	10.32	0.10	16.99	0.17
*m,p*-Xylene^b^	7.80	0.08	21.58	0.22
*o*-Xylene^a^	1.82	0.02	2.33	0.02
*o*-Xylene^b^	5.23	0.05	10.37	0.10
DMF^a^	13.34	0.44	60.57	2.02
DMF^b^	6.31	0.21	10.36	0.35
*Carcinogens*				
Chloroform^a^	11.33	2.61E-04	22.58	5.19E-04
Chloroform^b^	6.47	1.49E-04	12.18	2.80E-04
Ethylbenzene^a^	1.82	4.55E-06	5.32	1.33E-05
Ethylbenzene^b^	7.63	1.91E-05	20.35	5.09E-05

^a^Exposed area; ^b^Controlled area

## Discussion

Air quality monitoring is the main source of information for assessing the exposure of the population to ambient air pollution. Positive mutual correlations were significant among toluene, DMF, chloroform, and MEK in indoor, outdoor and personal exposure measurements. Benzene, ethylbenzene, *m,p*-xylene, and *o*-xylene were also mutually positively correlated in indoor, outdoor and personal exposure measurements. The high correlations between each other can be explained that the toxic pollutants could have come from the same source [20]. However, the correlation relationship was not found between the two groups. It can be implied that the two group VOCs might be emitted from different kinds of pollutant sources.

The data from PRTR of the Korean pollutant emissions showed that most of toluene, DMF, MEK, and chloroform were emitted from DDIC. Outdoor and indoor concentrations of toluene, DMF, and chloroform in the exposed area were significantly higher than the concentrations in the controlled area both in summer and winter. Toluene and chloroform were widely utilized in dyeing, finishing processes and warranting examination, and were markers of gasoline vehicle emissions [10, 17]. Benzene was not utilized in dyeing and finishing processes, it has been known as markers of gasoline vehicle emissions [18]. The concentrations of toluene, DMF, chloroform, and MEK in residential outdoor significantly decreased with increasing distances from DDIC. Conversely, the concentration of outdoor benzene was found to increase with distances increased from DDIC. Therefore, it may be suggested that toluene, DMF, chloroform, and MEK in the ambient air of the exposed area were mainly emitted from DDIC.

Benzene, ethylbenzene, *m,p*-xylenes, and *o*-xylenes were the major VOCs for mobile sources in the urban air [17]. The previous study also suggested that these compounds could have originated from the use of solvents and evaporative losses of fuel [10]. Several studies suggested that good correlations among benzene, toluene, ethylbenzene, and xylene from traffic emissions were *r* = 0.79–0.92 (*p* < 0.01) [21–23]. However, poor correlations of benzene, toluene, ethylbenzene, and xylene were observed (*r* = 0.14 ~ 0.76) in this study. This can be explained by the ethylbenzene and xylene that could have come from industrial solvents and traffic emissions, while benzene mainly came from traffic sources. The poor correlation could be also explained by the mixed sources of ethylbenzene and xylene in the exposed area.

The indoor, outdoor and personal exposure concentrations of toluene, DMF and chloroform in the exposed area were also significantly higher than the corresponding concentrations in the controlled area in summer and autumn. Positive mutual correlations were significant among toluene, DMF, chloroform, and MEK in indoor, outdoor and personal measuring. Benzene, ethylbenzene, *m,p*-xylene, and *o*-xylene were also mutually positively correlated in indoor, outdoor and personal measurements. The indoor and personal concentrations were all in accordance with the outdoor situations. Residential indoor sources, such as cooking, cigarette smoking, materials used on the wall flooring and furniture, could be major contributors to the indoor concentrations of VOCs. However, it can be suggested that the personal and indoor concentrations of selected VOCs were also obviously affected by outdoor pollutant sources in exposed area in DDIC. Personal exposure concentrations of VOCs were also affected by many factors including time-activity patterns. Even so, outdoor pollutant sources from DDIC affected the indoor air quality and personal exposure in the exposed area. Since VOCs easily evaporate or sublimate into the air at normal room temperature, they can migrate from a source and enter buildings nearby to a dangerous level [24]. It also differentiates the pollution amount of indoors by distances from emission sources [4].

Cancer risk for chloroform in the exposed area was greater than in the controlled area and even greater than 10^− 4^. HQ of toluene, DMF, and MEK in the exposed area were much higher than in the controlled area. Morbidities of respiratory diseases, anaphylactic diseases and cardiovascular diseases in the exposed area were significantly higher than in the controlled area. It also suggested that the health of residents living near DDIC could be affected by pollutants emitted from DDIC. The results suggested a need for environmental policies to reduce pollution and the DDIC residents exposure. The health assessment studies identified self-reported symptoms of asthma in both adults and children as a significant finding. It is appropriate for this health risk assessment to consider the mixture effects of the pollutants that are recognized respiratory irritants which are high enough in concentrations. The morbidities of asthma and other respiratory diseases in the exposed area were significantly higher than in the controlled area. The area nearby is a dense conglomerate of industry and human settlement. Special attention should be given to communities located in the areas close to DDIC.

Some VOCs concentrations such as toluene and MEK were higher in autumn than in summer. Concentrations in different seasons could be influenced by many factors, such as source variation, fuel consumptions, climatic conditions, and chemical reaction. Daegu has a continental climate with dry winters and hot summers. The area receives little precipitation except during the rainy season of summer. The data from Korea Meteorological Administration showed the average precipitation which varies from 224.0 mm to 235.9 mm between July and August (summer). On the contrary, the average precipitation varies from 33.8 mm to 30.5 mm between October and November (autumn). Average precipitation days (≥ 0.1 mm) varies from 14.4 days to 12.8 days between July and August, however, average precipitation days (≥ 0.1 mm) are approximately 5 days. The meteorological conditions make VOCs difficult to regional physical dispersion/ transportations and deposition. Another factor, that could have affected the concentration of VOCs in the summer, is dilution due to the increase in the mixing height [25]. Although the high temperature during summer will help the evaporation of VOCs, as they decay quickly by chemical removal reaction rates. Over the course of a year, the temperature of Daegu typically varies from − 5 °C to 32 °C. The daily mean temperature was 9.0 °C, and the average low temperature was 4.2 °C which is in November. After the middle of November, the indoor heating could have also contributed to the high concentrations of VOCs in autumn owing to fuel consumptions.

According to the study of Jo et al. (2004), the geometric mean concentration of toluene, benzene, *m,p*-xylene and *o*-xylene was 255 μg/m^3^, 7.2 μg/m^3^, 7.7 μg/m^3^ and 4.3 μg/m^3^ in outdoor for areas which are 100 m from the boundary of the DDIC, respectively, which was conducted between 10 October and 21 December 2000. As to the outdoor for areas between 500 m and 800 m away, the geometric concentration of toluene, benzene, *m,p*-xylene and *o*-xylene was 55.5 μg/m^3^, 10.5 μg/m^3^, 11.2 μg/m^3^ and 7.2 μg/m^3^, respectively [26]. In this study, the contemporaneous arithmetic mean value of toluene, benzene, *m,p*-xylene, and *o*-xylene was 161.37 μg/m^3^, 3.54 μg/m^3^, 10.48 μg/m^3^ and 1.73 μg/m^3^, respectively.

It was found out that VOCs can be emitted from many anthropogenic sources in urban and industrial areas, such as industrial processes and vehicle exhaust. Due to the obvious impacts on environments and human health, many studies suggested VOCs should be controlled for better regional air quality. However, there are few studies about specific industry VOC emission and impacts on the ambient environment [21, 27]. This study provided the detailed information about the ambient air concentrations of VOCs in nearby dyeing industry. Compared to similar studies, using a considerable large samples is the main strength of this study. Moreover, indoor, outdoor and personal exposure concentrations were measured both in the exposed area and the controlled area simultaneously, something that is rarely done in other studies.

The limitations of this study might be the possible confounding factors such as the distance from highly trafficked roads and indirect smoking. It was reported by 19.68% of volunteers in the exposed area and 10% in the controlled area that there were more than 6 lanes of roads within 500 m of their houses. And according to the study of Park et al. (2014), the concentrations of benzene, *m,p*-xylene, and *o*-xylene were influenced by exposure to second-hand smoke [28]. The data from the questionnaire showed that the rate of volunteers living with smokers was 13.98% and 12.98% in exposed area and the controlled area, respectively. The smoking of public places are forbidden in South Korea. Most participants responded that those living together did not smoke in the house indoors.

## Conclusions

Toluene, DMF, MEK, and chloroform in ambient air of exposed area were mainly emitted from DDIC. Health risks for toluene, DMF, MEK and chloroform in exposed area were significantly greater than in the controlled area. The prevalence of respiratory diseases, anaphylactic diseases and cardiovascular diseases in the exposed area were significantly higher than in the controlled area. This study showed that adverse cancer and non-cancer health effects may occur by VOCs emitted from DDIC, some risk managements are needed. Moreover, this study provides a preliminarily method for pollutants source characteristics.

## Abbreviations

DDIC: Daegu dyeing industrial complex
DMF: Dimethylformamide
HAPs: Hazardous air pollutants
HQ: Hazard quotients
IRIS: Integrated risk information system
LOD: Limit of detection (LOD)
MEK: Methyl ethyl ketone
MOE: Ministry of environment
PRTR: Pollutant release and transfer registers
R^2^: Coefficient of determination
VOCs: Volatile organic compounds
